# Can Gastric Juice Analysis with EndoFaster^®^ Reduce the Environmental Impact of Upper Endoscopy?

**DOI:** 10.3390/healthcare11243186

**Published:** 2023-12-17

**Authors:** Angelo Zullo, Federica Chiovelli, Enrica Esposito, Cesare Hassan, Beatrice Casini

**Affiliations:** 1 Gastroenterology Unit, “Nuovo Regina Margherita” Hospital, 00153 Rome, Italy; angelozullo66@yahoo.it; 2 Department of Translational Research and New Technologies in Medicine and Surgery, University of Pisa, 56126 Pisa, Italy; f.chiovelli@studenti.unipi.it (F.C.); 23706116@studenti.unipi.it (E.E.); 3 Gastroenterology and Endoscopy Unit, Department of Biomedical Sciences, IRCCS Humanitas Research Hospital, 20089 Milan, Italy; cesareh@hotmail.com

**Keywords:** CO_2_ emissions, carbon footprint, environmental impact, upper endoscopy, biopsies, EndoFaster^®^, gastric juice

## Abstract

Gastrointestinal (GI) endoscopy services are in third place as major contributors to CO_2_ emissions among healthcare facilities, especially due to their massive waste production. One of the measures suggested to reduce this environmental impact is a reduction in histological examinations performed on biopsy specimens taken during endoscopy. A reliable candidate to reduce the rate of biopsies and, consequently, the impact of CO_2_ emissions could be EndoFaster^®^, an innovative medical device that allows one to suspect or rule out both *H. pylori* infection and precancerous lesions on the gastric mucosa by analyzing a small amount of gastric juice aspirated during endoscopy in real time. In the present study, we investigated the ability of EndoFaster^®^ to reduce the environmental impact of upper endoscopy, comparing the CO_2_ production of standard biopsy sampling as suggested in guidelines and biopsies guided by real-time EndoFaster^®^ results during endoscopy. By estimating an overall 90% rate of biopsies according to standard guidelines and a reduction of 50% of gastric biopsies based on EndoFaster^®^ results, we calculated a 44% overall reduction in CO_2_ emissions, demonstrating that by using this tool, it is possible to distinctly reduce the contribution of upper endoscopy to global warming.

## 1. Introduction

Human activities are responsible for climate changes, which already have evident effects on the environment and human health [1]. The critical connection between human activities and environmental temperature increments is represented by greenhouse gases (GHGs) because of their impact on energy retention [2]. Up to 85% of all GHGs are represented by carbon dioxide (CO_2_). In order to indicate the total amount of CO_2_ equivalents released into the atmosphere as a result of the activities of an individual, a product, an institution, or a service, the term ‘carbon footprint’ is used.

The mission of healthcare organizations is to protect and enhance human health and well-being. However, it is estimated that healthcare activities have a remarkable carbon footprint, accounting for 1% to 5% of human environmental impact and about 4.4% of GHG emissions worldwide [3]. Among these activities, gastrointestinal (GI) endoscopy is reported as one of the largest polluters in terms of CO_2_ emissions and the third largest contributor to hazardous waste production in healthcare facilities, with about 3 kg of waste produced for each digestive endoscopy bed every day [4]. Therefore, different scientific societies, including the European Society of Gastrointestinal Endoscopy (ESGE), the European Society of Gastroenterology and Endoscopy Nurses and Associates (ESGENA), and the Italian Association of Hospital Gastroenterologists (AIGO), released specific documents aimed at outlining strategies to achieve a sustainable endoscopy practice, the so-called ‘Green Endoscopy’ [1,2].

One of the components that contributes the most to the high carbon footprint of digestive endoscopy is tissue sampling, which requires histological analysis because biopsy processing needs additional energy and generates hazardous waste [1,2]. Currently, in all the appropriate upper endoscopies, the standard gastric mucosa sampling consists of two biopsies on the antral (plus one on the *incisura angularis*) and two on the gastric body mucosa to be put in two different jars, which allows for the correct diagnosis of *H. pylori* infection and the disclosure of the presence and extension of precancerous lesions in the stomach [5,6]. *H. pylori* is the main cause of both benign (non-ulcer dyspepsia, peptic ulcer) and malignant (cancer, lymphoma) diseases, and the presence of diffuse precancerous lesions (atrophy, metaplasia) on the gastric mucosa distinctly increases the risk of cancer development [5,6]. It is widely reported that both of these conditions may be detected through histological assessment of biopsies taken on even normally appearing mucosa at white-light endoscopic examination [5,6,7,8,9]. Nevertheless, it is important to consider that routine histological analysis is unnecessary in a consistent percentage of patients with normal-appearing mucosa that eventually tests negative for both *H. pylori* infection and precancerous lesions. Indeed, the frequency of infection is relentlessly decreasing in developed countries, and the prevalence of diffuse gastric precancerous lesions is quoted as low as 3–7% in Western countries [10,11].

**Figure 1:**
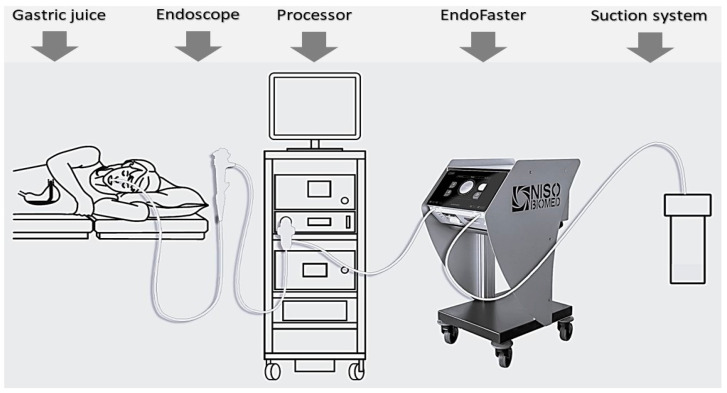
Gastric juice is aspirated during gastroscopy, passes through EndoFaster^®^, where it is analyzed in real time for both pH and ammonium concentration, and then it is discarded into the suction system.

In current European guidelines, upper endoscopy is suggested in patients with an increased risk of gastric cancer (patients >50 years old) and in those with alarm symptoms (bleeding, anemia, weight loss, persistent vomiting, dysphagia), while in dyspeptic, uninvestigated young patients, the ^13^C-Urea Breath Test (UBT), a less invasive alternative, should be preferred [5,6]. Nevertheless, even when upper endoscopy is appropriate, strategies for GHG emission reduction are needed, which can be summarized in the 3R principle (Reduce, Reuse, Recycle), with the reduction in waste and product generation as the top priority [1,2]. In GI endoscopy, by ensuring that only appropriate histological examinations are undertaken, it is possible to reduce the number of useless biopsy samples in low-risk patients without altering their management. Likewise, this approach is the most effective measure to lessen the impact on GHG emissions, according to the ESGE-ESGENA recommendations [1]. In line with this perspective, EndoFaster^®^ (producer: NISO Biomed, Turin, Italy; distributor: Waldner Tecnologie Medicali, Trento, Italy) may be a valid tool to select those patients who really require biopsy sampling of the gastric mucosa, avoiding inappropriate biopsies in low-risk patients and, consequently, reducing the environmental impact. Indeed, EndoFaster^®^ is an innovative, intuitive, and easy-to-use medical device that automatically analyzes a small (3 mL) amount of gastric juice aspirated during upper endoscopy, allowing one to suspect or rule out both *H. pylori* infection and diffuse precancerous lesions (atrophy with or without metaplasia) on the gastric mucosa in real time [12,13]. In detail, the machine is interposed between the endoscope and the suction system so that no adjunctive invasive procedure is required and there is no discomfort for the patient (Figure 1). The diagnosis is based on the determination of ammonium concentration and pH levels. The first is linked to the urease activity of the bacterium and provides information on the infection, while the latter allows the detection of hypochlorhydric conditions in gastric juice related to atrophy/metaplasia involving the gastric body mucosa.

Based on these considerations, we designed this study to evaluate the ability of EndoFaster^®^ to reduce the environmental impact of upper endoscopy, comparing the CO_2_ production of standard biopsy sampling performed in all patients as suggested in guidelines and biopsies guided by real-time EndoFaster^®^ results during endoscopy.

## 2. Materials and Methods

We estimated the CO_2_ production for either biopsy sampling guided by real-time EndoFaster^®^ results during endoscopy or standard biopsy sampling performed in all patients, as suggested in guidelines [5,6,7,8,9].

In detail, for the application of EndoFaster^®^ procedures, we first evaluated the daily CO_2_ production due to the energy consumed by the machine (51.04 Watts/day), taking as reference the data from an ISPRA report [14]. In addition, we considered the contribution of the following consumable materials with a relevant carbon component: (a) 3 bottles for calibration plus a liquid-draining system; (b) cardboard box for the 3 bottles; (c) washing solution tank; and (d) gastric juice suction tube. We did not include calibration liquids and reagents in this calculation, as they are highly diluted aqueous solutions that, in waste treatment plants, provide a positive contribution to the treatment process by helping to dilute solid waste without adding carbon. Furthermore, we computed the CO_2_ impact due to the disposal of liquid residues eliminated by the machine (5.308 L/day), transported by a 30,000 L chemical tanker traveling about 30 km between the hospital and disposal plant.

For the histological assessment, we also calculated the contribution of the following consumable materials: (a) biopsy forceps and (b) biopsy jar. In addition, we considered the entire biopsy processing in the pathology laboratory, including 11 steps (from specimen arriving in the laboratory to the pathologist’s review and report), accounting for 0.560 kg CO_2_/test, as accurately described elsewhere [15].

In order to calculate the CO_2_ production due to consumable material disposal, we used conversion factors adopted by the Institute for Sustainability Leadership of the University of Cambridge [16] and the IPCC Intergovernmental Panel on Climate Change [17]. In particular, the GHG emission factor from incineration of plastics was calculated at 2.697 kg CO_2_e/t of plastic waste using the formula kg CO_2_ = kg waste for incineration × oxidation factor of carbon in incinerator (0.98) × conversion factor of C to CO_2_ (3.67) × Σ(waste fraction (in %)) × dry matter content × carbon content (g/g dry weight). The dry matter content of plastic waste was equal to 1. The carbon content of plastic waste was 0.75 (Gg C/Gg dry weight waste). Moreover, the end-of-life emissions varied between different plastic types. The emissions for incineration, for instance, were higher for polystyrene (PS) and polyethylene (PE) (around 3 kg/kg plastic) and lower for polypropylene (PP) and polyurethane (PUR) (around 2.5 kg/kg plastic). For the purpose of this work, 2.7 kg CO_2_/kg plastic have been used for all incinerated end-of-life plastics.

## 3. Results

By hypothetically considering an endoscopy unit in which 2000 upper endoscopies are performed yearly, the CO_2_ production due to the energy consumed by the EndoFaster^®^ machine was estimated at 260.5 g CO_2_/kWh, corresponding to 21 kg CO_2_ /year (51.04 Watt × 8 h utilization/day × 200 days of use at full work/year). The contribution of consumable materials resulted in 50 kg/year (0.025 kg/test), a value conservatively calculated without considering the portion of materials that can be recycled (Table 1). For the disposal of liquid residues produced by the machine, we computed 0.98 kg/year (assuming 2.62 kg CO_2_/L of diesel).

For standard biopsy sampling, with the use of one biopsy forceps and two jars with formaldehyde, the production of CO_2_ resulted in 1262 kg/year (0.70 kg/test) following the entire process up to the histological report (Table 1).

Thus, by estimating an overall 90% rate of biopsies, according to what is advised by current guidelines to perform a high-quality upper endoscopy, and a reduction of 50% of gastric biopsies, based on the EndoFaster^®^ results, the yearly CO_2_ production would be 704 kg instead of 1262 kg, accounting for an overall 558 kg CO_2_ reduction (44%) (Table 2).

**Figure 2:**
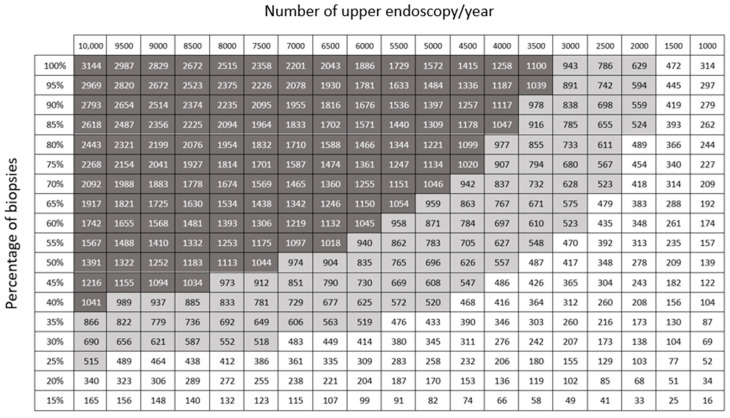
Reduction in yearly CO_2_ production (kg) by using EndoFaster^®^ according to the number of endoscopies and rate of standard biopsies performed. By considering values >1500, 1500–500, and <500 (arbitrarily chosen), the reduction could be considered high, intermediate, and low, respectively.

Since the number of endoscopic examinations performed yearly and the rate of standard biopsies taken during endoscopy may vary in different centers [18], we constructed a nomogram to calculate various scenarios of CO_2_ reduction by using EndoFaster^®^ (Figure 2).

## 4. Discussion

A large amount of carbon is emitted by healthcare institutions. Between 2000 and 2019, there was an annual increase of 1.6% in energy usage, according to the Energy Statistics Handbook (2021). Therefore, carbon footprint represents an important area of interest for sustainable healthcare in the future [19].

Within the healthcare field, GI endoscopy is a larger contributor to the carbon footprint than other fields because it is associated with high daily caseloads, the production of high-volume non-renewable waste, the utilization of single-use devices, reprocessing or decontamination processes, and repetitive travel by patients and their families [19]. In the USA, the impact of endoscopy was estimated at 85,768 metric tonnes of CO_2_ emissions annually, corresponding to >9 million gallons of gasoline consumed, 94 million pounds of coal burned, and 212 million miles driven in an average non-electric car [20].

A significant contribution to the high environmental impact of digestive endoscopy comes from the routine histological analysis of biopsy specimens taken during the endoscopy procedure. This has been clearly demonstrated in the study from which we derived the CO_2_ amount produced during the entire biopsy processing in the pathology laboratory, which showed that the environmental impact of one cassette processed by the pathology laboratory corresponds to 0.28 kg of carbon dioxide equivalents (CO_2_e) per examination in the case of one biopsy jar and 0.79 kg of CO_2_e in the case of three jars, corresponding to a car with a passenger traveling 0.7 and 2 miles, respectively. By applying this to more than 20 million biopsies performed in the US annually, it turns out that emissions from biopsy processing are equivalent to the yearly GHG emissions from 1200 passenger cars [15]. Another study demonstrated that just placing small tissue samples obtained from polypectomy collectively in a single specimen pot would result in a reduction in carbon footprint equivalent to 396 kg CO_2_e (emissions from 982 miles driven by an average passenger car) [21].

The EndoFaster^®^ tool has been proven by previous studies to enable a significant reduction in gastric biopsies, with consequent decreased costs (biopsy forceps, formalin vials, histologic preparation, histologic analysis, etc.) and important health resource savings [22]. The data from the present study found that EndoFaster^®^ could also distinctly reduce the environmental impact of upper endoscopy. Indeed, we calculated that through the application of this technology, it is possible to obtain a reduction of 44% in the yearly CO_2_ production of upper endoscopy in a center hypothetically performing 2000 endoscopies a year with an estimated 90% rate of biopsies, as advised by current guidelines to perform a high-quality upper endoscopy [9]. In detail, by using the device, a reduction of 558 kg of CO_2_ is expected. By considering that a tree absorbs an average of 22 kg of CO_2_ per year, the use of EndoFaster^®^ would correspond to planting 27 trees around an endoscopy center that performs 2000 endoscopies per year. Of note, this reduction can be obtained without losing clinically relevant information for the patients, since EndoFaster^®^ excludes the presence of both *H. pylori* infection and diffuse precancerous lesions in the stomach with negative predictive values (NPVs) as high as 97–98% in a population with a low prevalence of these conditions [12,13]. This would mean that only 2–3 of every 100 patients classified as negative by the test would eventually be infected or have a precancerous lesion, even lower when considering that some false-negative results for one finding (i.e., pH results) could be recovered by a positive result at the other finding (i.e., ammonium concentrations).

The reduced environmental impact achievable through the use of EndoFaster^®^ compared to standard biopsy sampling performed in all patients further supports the possibility of using this technology to minimize the use of histology in appropriate clinical pathways. Indeed, in the majority of patients with normal-appearing gastric mucosa who eventually test negative for both *H. pylori* infection and precancerous lesions, routine histological analysis is substantially useless [10]. Thus, a strategy that allows for the avoidance of inappropriate and invasive biopsies, identifying patients who really need biopsy sampling and those who do not, also reduces CO_2_ emissions, proving advantageous.

To the best of our knowledge, the present study is the first to demonstrate how the possibility of avoiding foreseeable negative gastric biopsies in a definite portion of patients through the application of EndoFaster^®^ could distinctly reduce the environmental impact of upper endoscopy.

## 5. Conclusions

Healthcare processes are among the activities producing GHGs, so more efforts within the healthcare community are needed to promote environmental sustainability by reducing healthcare-associated emissions and implementing energy-efficient practices. Undeniably, GI endoscopic procedures are among the highest generators of waste, mainly due to the employment of several single-use devices. One of the activities that implies a major consumption of materials, considering the high rate of endoscopic examinations performed yearly, is the histological assessment of the mucosa. Furthermore, histological analysis is frequently negative in several patients, which implies a useless consumption of healthcare resources and avoidable CO_2_ emissions. Thus, technologies that minimize the use of histology should be implemented within appropriate clinical pathways from the perspective of a Green Endoscopy.

## Figures and Tables

**Table 1:** CO_2_ production for consumable materials of EndoFaster^®^ and standard biopsy sampling.

Material	Type	Quantity (g)	Waster Disposal	Quantity/ Test	kg CO_2_/ kg Waste	kg CO_2_/ Test
Bottle 1 for calibration + liquid-draining system	Polyethylene (PE)	78.6	Plastic recycling	1.31	3.0	0.004
Bottle 2 for calibration + liquid-draining system	PE	51.6	Plastic recycling	0.86	3.0	0.003
Bottle 3 for calibration + liquid-draining system	PE	51.6	Plastic recycling	0.86	3.0	0.003
Cardboard box for the 3 bottles	Carton	137	Cardboard recycling	2.28	0.95	0.002
Washing solution tank	High-density polyethylene (HDPE)	146	Plastic recycling	2.43	3.0	0.007
Gastric juice suction tube	Plastic (mixed)	25	Infected waste	2.08	3.0	0.006
Biopsy forceps	Plastic (mixed)	20	Infected waste	1	3.0	0.060
Biopsy jar	Plastic	-	Infected waste	2	3.0	0.081

**Table 2:** Estimation of yearly CO_2_ reduction by performing biopsies based on EndoFaster^®^ results.

Total procedures/year	2000
Standard biopsy sampling	90%
Procedures with biopsy sampling	1800
Reduction in biopsies through the use of EndoFaster^®^	50%
Biopsy sampling avoided	900
kg CO_2_ produced by EndoFaster^®^ materials	50
kg CO_2_ produced by EndoFaster^®^ due to energy consumption and liquid residue disposal	22
kg CO_2_ total normally produced (without biopsy reduction)	1262
kg CO_2_ total saved yearly through the use of EndoFaster^®^	630
kg CO_2_ reduction	558
Percentage of CO_2_ reduction	44%

## Data Availability

Data are contained within the article.
